# Data Disclosure for Chemical Evaluations

**DOI:** 10.1289/ehp.1204942

**Published:** 2012-12-11

**Authors:** Randall Lutter, Craig Barrow, Christopher J. Borgert, James W. Conrad, Debra Edwards, Allan Felsot

**Affiliations:** 1 Independent Consultant, Bethesda, Maryland, USA; 2 Craig Barrow Consulting, Gibsonia, Pennsylvania, USA; 3 Applied Pharmacology and Toxicology Inc., Gainesville, Florida, USA; 4 Conrad Law & Policy Counsel, Washington, DC, USA; 5 Independent Consultant, Alexandria, Virginia, USA; 6 Food and Environmental Quality Lab, Washington State University, Richland, Washington, USA

**Keywords:** chemicals, data disclosure, information quality, pesticides

## Abstract

Background: Public disclosure of scientific data used by the government to make regulatory decisions for chemicals is a practical step that can enhance public confidence in the scientific basis of such decisions.

Objectives: We reviewed the U.S. Environmental Protection Agency’s (EPA) current practices regarding disclosure of data underlying regulatory and policy decisions involving chemicals, including pesticides. We sought to identify additional opportunities for the U.S. EPA to disclose data and, more generally, to promote broad access to data it uses, regardless of origin.

Discussion: We recommend that when the U.S. EPA proposes a regulatory determination or other policy decision that relies on scientific research, it should provide sufficient underlying raw data and information about methods to enable reanalysis and attempts to independently reproduce the work, including the sensitivity of results to alternative analyses. This recommendation applies regardless of who conducted the work. If the U.S. EPA is unable to provide such transparency, it should state whether it had full access to all underlying data and methods. A timely version of submitted data cleared of information about confidential business matters and personal privacy should fully meet the standards of transparency described below, including public access sufficient for others to undertake an independent reanalysis.

Conclusion: Reliable chemical evaluation is essential for protecting public health and the environment and for ensuring availability of useful chemicals under appropriate conditions. Permitting qualified researchers to endeavor to independently reproduce the analyses used in regulatory determinations of pesticides and other chemicals would increase confidence in the scientific basis of such determinations.

The evaluation of chemicals is an important topic of public interest. Against this backdrop, CropLife America (Washington, DC), an association of agricultural pesticide manufacturers, sponsored a meeting of experts from a variety of backgrounds to address how to judge the quality of scientific work in chemical evaluation and, if possible, to seek consensus or agreement. Here we present a proposal from some of those experts addressing a more specific topic: disclosure of and access to data underlying regulatory determinations concerning pesticides and other chemicals.

It is axiomatic that scientific work used in regulatory determinations should be of high quality [e.g., Information Quality Act (IQA) 2000]. Greater public disclosure of data and methods is a practical step toward ensuring that scientific work used in regulatory determinations meets this standard for quality. Greater disclosure should reduce bias because it makes masking of bias more difficult. In addition, the reliability of scientific work used in regulatory evaluations of chemicals is likely to improve if greater disclosure leads to increased evaluation of data quality and that evaluation then leads to improved designs and generally higher-quality studies. Furthermore, access to the underlying raw data and methodology may be required for the public to provide more informed comments to regulatory agencies that will rely on the study (Portland Cement Association *v.* Ruckelshaus 1973). Ultimately, the reliability of scientific work can be judged definitively only if researchers have disclosed sufficient data and information about methods and results to permit others to evaluate data quality and to try to reproduce or replicate key findings, including the sensitivity of results to alternative analyses.

This does not mean that independent replicability is by itself a standard sufficient for quality. Replicability by independent entities is one of the three generally accepted tenets of valid regulatory science. The other two tenets are that the identity and authenticity of scientific measurements be verifiable within a defined range of precision, and that measurements and observations not be confounded by extraneous factors known to corrupt their accuracy and precision (Borgert et al. 2011; Gori 2009a, 2009b). The heads of the National Institute of Environmental and Health Sciences and the Agency for Toxic Substances and Disease Registry have endorsed these tenets in testimony (Birnbaum and Falk 2010). Henry and Conrad (2008) discussed a variety of other standards and practices (e.g., peer review) that can be used as indications of quality. Although disclosure by itself may not be sufficient to ensure quality, it is necessary.

The IQA requires the U.S. Office of Management and Budget (OMB) and agencies such as the U.S. Environmental Protection Agency (EPA) to issue guidelines for ensuring and maximizing the quality, objectivity, utility, and integrity of information disseminated by federal agencies. The OMB’s guidelines under the IQA embrace a disclosure principle, stating that when agencies disseminate “influential scientific, financial, or statistical information,” they “shall include a high degree of transparency about data and methods to facilitate the reproducibility of such information by qualified third parties” (OMB 2002). The OMB guidelines explain that the standard they use, “capable of being substantially reproduced,” is less stringent than a “confirmation” standard because it simply requires that an agency’s analysis be sufficiently transparent that another qualified party could replicate it through reanalysis. The U.S. EPA has its own information quality guidelines, which are consistent with those of the OMB (U.S. EPA 2002). Although one federal district court found that the IQA “does not create a legal right to access to information” (Salt Institute *v.* Leavitt 2006), two more recent federal appeal courts left open the possibility of judicial review of agency actions measured against IQA requirements (Americans for Safe Access *v.* U.S. Department of Health and Human Services 2010; Prime Time International Co. *v.* Vilsack 2010). Thus, the IQA at a minimum provides support for the disclosure concepts discussed here and may provide opportunities for enforcing such disclosure.

Legislation commonly known as the “Shelby Amendment” (Treasury and General Government Appropriations Act 1998) led the OMB to revise its Circular A-110 so that, in response to a Freedom of Information Act (FOIA 1966) request, federal agencies must release research data relating to published research findings produced under an award (e.g., federal grant or contract) that were considered by the agency in developing action that has the force and effect of law (American Association for the Advancement of Science 2005; OMB 1999). Much of the research that the U.S. EPA relies on in making decisions regarding regulated chemicals, particularly pesticides, is not federally funded, although published studies cited in the U.S. EPA’s Integrated Risk Information System (IRIS) often received federal funding.

In describing implementation of the Shelby Amendment, Conrad and Becker (2011) stated that

[i]t seems only fair for privately-funded work to be subject to the same disclosure requirement, at least when the persons conducting or funding it submit it to an agency.

Similarly, in their report *Improving the Use of Science in Regulatory Policy*, the Bipartisan Policy Center (2009) recommended that

Studies used in the formulation of regulation should be subject to data access requirements equivalent to those under the Shelby Amendment and its implementing Circular regardless of who funded the study.

Several prominent journals have adopted data disclosure policies intended to facilitate replication. *Nature*’s policy (Nature Publishing Group 2012) states:

An inherent principle of publication is that others should be able to replicate and build upon the authors’ published claims. Therefore, a condition of publication in a *Nature* journal is that authors are required to make materials, data and associated protocols promptly available to readers without undue qualifications.

Similarly, the policy of the *Proceedings of the National Academy of Sciences* (PNAS 2012) states: “To allow others to replicate and build on work published in *PNAS*, authors must make materials, data, and associated protocols available to readers.” *Science* has similar policies (Science 2012) and recently published a special section on the importance and challenges of data replication and reproducibility in different fields (Jasny et al. 2011).

Our recommendations are consistent with the Shelby Amendment, recommendations of the Bipartisan Policy Center, and the practices of prominent journals, as well as the recommendations of Conrad and Becker. Our goal is to promote the broadest possible access to data used by the U.S. EPA, regardless of who prepared or compiled the data.

## Discussion

The U.S. EPA already has access to considerable data underlying studies submitted by pesticide registrants that it uses in regulatory decisions regarding pesticides. For example, if a regulated entity submits to the U.S. EPA a Good Laboratory Practice (GLP) study (Good Laboratory Practice Standards 1989) required for a pesticide registration, it must retain all “raw” data to comply with GLP requirements (e.g., 40 CFR 160.190 and 40 CFR 160.195; U.S. EPA 1989). The U.S. EPA has access to such data because, for the purpose of supporting a pesticide registration, it may refuse to consider reliable any data from a study that is not conducted in accordance with those GLP rules (40 CFR 160.17). On the other hand, a U.S. EPA request for data used in a peer-reviewed or “gray literature” study may be fulfilled completely, partially, or not at all.

We recommend that when the U.S. EPA proposes a regulatory determination or other policy decision for pesticides or other chemicals that relies on scientific research, it should provide sufficient disclosure of data and information about methods to enable reanalysis and attempts at independent replication of the work, including the sensitivity of results to alternative analyses. This recommendation applies whether the decision is a discrete compound-specific decision, such as setting an uncertainty factor or determining a benchmark dose, or a programmatic policy decision, such as adoption of a particular study design or method for particular types of testing. Such disclosure should include all raw data—that is, data as originally collected in accordance with research protocols, the research protocols themselves, and all methods (including computer programs used for statistical modeling). Thus it would extend from the supporting science (e.g., animal toxicity studies used to calculate cancer slope factors) to risk assessments (i.e., analytic work that takes as given the results of toxicological and epidemiological work and integrates them into an assessment of risk). The recommended disclosure would be sufficiently detailed to include recorded ages and sex of test animals, all laboratory results, and all recorded observations about health and clinical conditions, with all disclosed data recorded according to research protocols. Disclosure should be sufficient to provide for a full understanding of the operation of any proprietary models used in supporting studies.

Further, this recommendation applies regardless of who conducted the work (e.g., researchers with industry, government, or academic institutions). In instances where the U.S. EPA is unable to provide such a level of transparency because of lack of access or legal restrictions on disclosure, it should state the degree of access it had to such data. Finally, if the U.S. EPA did not enjoy full access, it should offer a cogent explanation of why it decided to make regulatory or policy decisions using results of analyses that lacked the ideal level of transparency and how, specifically, it weighed such results relative to other evidence.

The U.S. EPA has taken some constructive steps in this direction. Its Office of Pesticide Programs issued *Evaluation Guidelines for Ecological Toxicity Data in the Open Literature* (U.S. EPA 2011) that partially implements the ideas discussed here. In particular, the guidelines (U.S. EPA 2011) acknowledge that the “most reliable means of determining whether study conclusions can be verified is through access to the raw data” and state that

[w]here raw data are not available to verify the study endpoints, the reviewer must discuss the uncertainties associated with quantitative use of the data relative to studies where raw data are provided.

Finally, the U.S. EPA (2011) advised analysts that

[d]epending on the importance of the open literature study to the risk assessment conclusions, attempts should be made to obtain missing information from the study, including the raw data, if possible.

Although these steps represent improvements in access to and disclosure of underlying data, they fall short of our recommendations. First, they do not apply generally to all data used for chemical evaluation. Second, the U.S. EPA (2011) guidelines discuss access to raw data as important for verification of conclusions. However, there is no mention of replication of results, although replication (including an assessment of the robustness of results) is an essential part of ensuring validity. In addition, these U.S. EPA guidelines are silent about access to detailed information about methods (e.g., computer code). Fourth, the guidelines require an analyst to “discuss the uncertainties associated with quantitative use of the data.” A better approach, adopted here, would be for the U.S. EPA to state that it will explain how, specifically, it weighed such results relative to other data. Finally, the guidelines (U.S. EPA 2011) limit instructions to obtain raw data “depending on the importance of the open literature study,” and appear focused on “missing information” instead of declaring that all raw data underlying studies used in quantitative regulatory determinations should be generally available to the U.S. EPA and the public.

Our recommendation does not mean that the U.S. EPA should require that all disseminated data be subjected to a reproducibility requirement. As explained in the OMB information quality guidelines (OMB 2002), constraints related to ethics, feasibility, or confidentiality may preclude disclosure or a replication exercise (i.e., a new experiment, test, or sample) prior to each dissemination. Instead, we recommend that the U.S. EPA generally provide sufficient transparency about data and methods that a qualified member of the public could undertake an independent reanalysis. These standards for transparency should apply to agency analyses of data from a single study as well as to analyses that combine information from multiple studies.

Section 10 of the Federal Insecticide, Fungicide, and Rodenticide Act (FIFRA 1972) provides for public access to safety and efficacy information (U.S. EPA 2010). There are two types of exceptions, which are important to respect and which have been implemented without undermining the objectives of disclosure discussed here. First, certain information that is generally not related to assessing risks or making regulatory determinations is excluded from disclosure as confidential business information. By law, the U.S. EPA may not make public information that discloses *a*) manufacturing or quality control processes, *b*) methods for testing and measuring the quantity of deliberately added inert ingredients, and *c*) the identity or percentage quantity of deliberately added inert ingredients (FIFRA 1972). [We note that on 23 December 2009 the U.S. EPA issued an advance notice of proposed rule making to increase the public availability of information regarding the identity of the inert ingredients of pesticide products (U.S. EPA 2009).]

Second, FIFRA protects the proprietary interests of the pesticide manufacturers that first made the investments necessary to produce the data by requiring the U.S. EPA to ensure that the release of data does not unfairly benefit the competitors of those companies (FIFRA 1972). To accomplish this, the U.S. EPA must obtain—before disclosure of such data—affirmations from recipients that they will not give the data to multinational business interests that might seek to register in other countries the pesticide products that are the subject of the testing (U.S. EPA 2012a). In addition, the agency must keep lists of the people who obtain such data and who they represent.

The U.S. EPA currently reviews and redacts data before a version cleared of confidential business information (CBI) can be made public. This process currently requires the public to file a formal request under FOIA for each study for which it wants undisclosed information. The U.S. EPA reported to Congress in 2010 that it has “completely redesigned its electronic FOIA reading room to make tens of thousands of highly sought after pesticide science and regulatory records publicly available without the filing of a FOIA request” (Gottesman 2010). To further advance such reforms, we suggest that the U.S. EPA convene a diverse stakeholder group (e.g., through its Pesticide Program Dialogue Committee; U.S. EPA 2012b) to solicit specific ideas about ways to streamline the current process to facilitate timely disclosure of data consistent with legal protections under FIFRA and FOIA. A timely CBI-cleared version of industry-submitted data should fully meet the standards of transparency described here, including public access to enough data and details of the study design that others could undertake an independent replication effort.

The timing of data disclosure matters. The U.S. EPA should make publicly available data underlying a regulatory determination or other policy decisions for pesticides by the beginning of the applicable public comment period to provide interested members of the public a meaningful opportunity for review before commenting on the proposal. Disclosure would generally occur after publication of academic articles. An exception would occur if the publication process was unavoidably so lengthy that the study was forthcoming rather than published when used by the regulator in a proposed regulatory or policy decision. If the agency uses data submitted by a manufacturer that are protected from release by federal law, the regulatory agency should provide information on the data and methods generally in a manner that facilitates efforts at independent analysis by qualified members of the public.

## Conclusion

Evaluating chemicals within a science-based framework is essential to protecting public health and the environment and ensuring availability of useful chemicals under appropriate terms and conditions. Public access to data and methodologies used in regulatory determinations is equally essential to maintaining public trust in regulators’ decisions. The principles and recommendations we describe here regarding data access will help achieve these goals by permitting qualified researchers to endeavor to replicate analytic results independently.
